# Risk Factor Analysis for Growth Arrest in Paediatric Physeal Fractures—A Prospective Study

**DOI:** 10.3390/jcm13102946

**Published:** 2024-05-16

**Authors:** Nikki Hooper, Liam Johnson, Nicole Banting, Rubini Pathy, Emily K. Schaeffer, Jeffrey N. Bone, Bryn O. Zomar, Ash Sandhu, Caitlyn Siu, Anthony P. Cooper, Christopher Reilly, Kishore Mulpuri

**Affiliations:** 1 Department of Orthopaedic Surgery, University of Otago, Christchurch 9016, New Zealand; 2 Department of Orthopaedic Surgery, Queensland Children’s Hospital, Brisbane, QLD 4101, Australia; 3 Department of Orthopaedic Surgery, BC Children’s Hospital, Vancouver, BC V6H 3N1, Canada; 4 Department of Orthopaedics, Alberta Children’s Hospital, Calgary, AB T3B 6A8, Canada; 5 Department of Orthopaedics, University of British Columbia, Vancouver, BC V5Z 1M9, Canada; 6 Department of Obstetrics and Gynaecology, BC Children’s Hospital, Vancouver, BC V6H 3N1, Canada

**Keywords:** growth arrest, trauma, humerus, radius, ulna, femur, tibia, fibula, limb length discrepancy, Salter–Harris, fracture, angular deformity

## Abstract

**Background:** Fractures through the physis account for 18–30% of all paediatric fractures, leading to growth arrest in up to 5.5% of cases. We have limited knowledge to predict which physeal fractures result in growth arrest and subsequent deformity or limb length discrepancy. The purpose of this study is to identify factors associated with physeal growth arrest to improve patient outcomes. **Methods:** This prospective cohort study was designed to develop a clinical prediction model for growth arrest after physeal injury. Patients ≤ 18 years old presenting within four weeks of injury were enrolled if they had open physes and sustained a physeal fracture of the humerus, radius, ulna, femur, tibia or fibula. Patients with prior history of same-site fracture or a condition known to alter bone growth or healing were excluded. Demographic data, potential prognostic indicators, and radiographic data were collected at baseline, during healing, and at one- and two-years post-injury. **Results:** A total of 332 patients had at least six months of follow-up or a diagnosis of growth arrest within six months of injury. In a comparison analysis, patients who developed growth arrest were more likely to be older (12.8 years vs. 9.4 years) and injured on the right side (53.0% vs. 45.7%). Initial displacement and angulation rates were higher in the growth arrest group (59.0% vs. 47.8% and 47.0% vs. 38.8%, respectively), but the amount of angulation was similar (27.0° vs. 28.4°). Rates of growth arrest were highest in distal femoral fractures (86%). **Conclusions:** The incidence of growth arrest in this patient population appears higher than the past literature reports at 30.1%. However, there may be variances in diagnostic criteria for growth arrest, and the true incidence may be lower. A number of patients were approaching skeletal maturity, and any growth arrest is likely to have less clinical significance in these cases. Further prospective long-term follow-up is required to determine risk factors, incidence, and true clinical impact of growth arrest when it does occur.

## 1. Introduction

Fractures are a common complication of childhood with a cumulative risk of paediatric fracture of 27% for girls and 42% for boys [1]. Children’s bones differ from adult bones in that they are still growing. Growth takes place at the physis, a layer of cells that continue to reproduce and turn into bone. The growth plate determines the length and shape of the adult bone.

Fractures through the physis account for 18–30% of paediatric fractures and can lead to growth arrest (GA) in up to 5.5% of cases [2,3,4,5,6]. To put this in perspective, based on the 2009 population of children in British Columbia of 971,940 [7], approximately 81,022 children would have a fracture involving the growth plate during his or her childhood. Of these children, approximately 4456 would be expected to experience GA.

Unfortunately, we have limited knowledge to predict which physeal fractures will result in GA and subsequent deformity and the incidence is variable depending on the location, type of injury and management [4,5,8,9]. Premature GA is characterised by unexpected cessation of longitudinal growth due to an insult, while the physis remains open and can either affect the entire physis, resulting in shortening of the limb, or part of the physis, causing an angular deformity [9,10]. These complications may have a long-term impact on a child’s life by causing osteoarthritis, difficulty walking, or spine disorders [11]. Previous studies have examined associations between patient demographics, fracture characteristics, and severity of injury with the incidence of GA but have been limited by retrospective study designs or small sample sizes [2,3,4,8].

Although retrospective cohort or case–control designs are excellent options for studying rare events such as GA, a significant disadvantage of a retrospective series is that certain potentially important factors may not be recorded or available through a chart review, retrospective interview, or follow up exam. Factors which may be of importance, but which may not be sufficiently documented, include accurate bone age at the time of injury, number of hours to definitive treatment, the exact area of the growth plate involved, and details on the energy and mechanism of injury. One strength of a prospective cohort study design is the ability to decrease bias by measuring the confounders that we know about.

A better understanding of factors associated with physeal GA will improve patient care and has the potential to impact health resource utilization. The ability to predict physeal GA in long bone fractures would be beneficial for patients. Limb length discrepancies can be more easily treated with early detection and intervention, resulting in a less invasive surgery [12,13]. If detected later when there is less, or no growth remaining for growth velocity manipulation, a larger and more invasive surgery is often necessary, such as osteotomy or distraction osteogenesis.

In addition, clarifying prognostic factors may allow surgeons to make more appropriate follow-up care plans. Currently, children who experience physeal fractures follow-up with the surgeon two or three times after the fracture has healed to monitor growth for signs of GA. If we had a tool for prognosticating GA, this may allow low-risk patients to avoid additional clinical and radiographic visits, and high-risk patients may be seen at an appropriate level of priority. The ability to discern which children are at risk of GA could lead to more appropriate utilization of health resources such as clinic time and radiograph use. The purpose of this study is to identify factors associated with premature GA after trauma to improve patient outcomes and to report the rate of GA in a prospectively collected cohort.

## 2. Materials and Methods

### 2.1. Patient Population

This prospective cohort study was designed to observe rates of GA following physeal fractures and examine clinical predictive factors in skeletally immature patients. Patients aged 18 years or younger, who presented within four weeks of injury at our tertiary paediatric hospital, were enrolled if they had open physes and sustained a physeal fracture of the humerus, radius, ulna, femur, tibia or fibula. Patients with a previous fracture at the same site or a condition known to alter bone growth or healing were excluded. Demographic data, potential prognostic indicators and radiographic data were collected at baseline, during healing and at one- and two-years post-injury.

### 2.2. Data Collection

All patients were managed by one of seven paediatric orthopaedic surgeons and treated according to best clinical practice in line with the responsible surgeon’s preference. Initial displacement in millimetres and angular deformity at presentation were recorded. Reduction attempts and methods were noted, as well as any required surgical intervention. Follow up radiographs were performed post-reduction, at 6–8 weeks, 4–8 months, 1 year and 2 years post-injury. All films were assessed for residual deformity (both displacement and angulation) and any signs of a physeal bar or premature physeal closure. All initial radiographs were classified according to the Salter–Harris (SH) classification system [9]. Comparison radiographs of the contralateral side were obtained at 1- and 2-year follow-up visits when possible and used for further assessment. All participants were reviewed by two independent, blinded, fellowship-trained paediatric orthopaedic surgeons (NH and LJ) for both SH classification on initial films and any evidence of GA at final follow-up. Evidence of GA included any physeal bar formation, premature closure on comparison contralateral views or growth disturbance with resultant angular deformity. In cases of discrepancies in opinion, a third paediatric orthopaedic surgeon (KM) was involved, and a consensus was obtained.

### 2.3. Data Analysis

All data were summarised as means and standard deviations for continuous variables and counts and percentages for categorical variables. The agreement in radiograph data was summarised with Cohen’s Kappa statistic with a 95% bootstrap confidence interval based on 1000 resamples. To determine possible risk factors for growth arrest, univariate and multivariable logistic regression models were fit and both crude and adjusted odds ratios, 95% profile confidence intervals and Wald *p*-values are reported. We assessed the following four primary risk factors that were selected a priori based on clinical relevance: Salter–Harris Classification, age at baseline, requirement of reduction and requirement of operative fixation. Adjusted results came from a multivariable model including each of these variables as well as fracture location and patient sex. There were very few cases (<5) of missing data on these predictors, so only a complete case analysis was conducted. Finally, we assessed the relationship between detailed location of injury (fracture location + distal or proximal) using a univariate logistic regression model with ‘distal radius’ (the largest group) as the reference category. Due to sample size limitations, we did not adjust this model for other variables. All analyses were carried out using R version 3.5.3 [14].

## 3. Results

**Figure 1:**
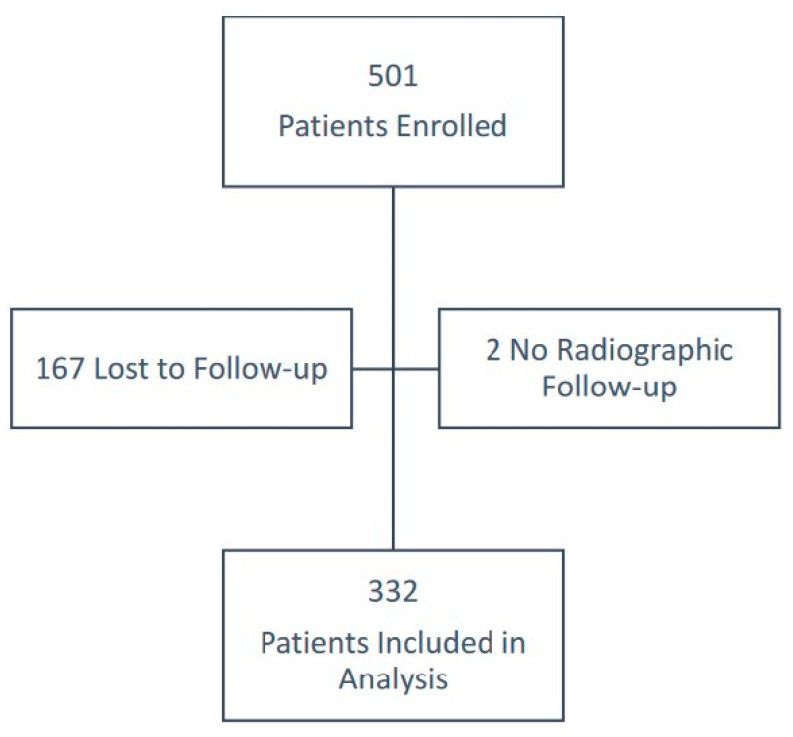
Flow diagram of participation in the study.

There was a total of 501 patients who presented with a physeal fracture of a long bone between September 2011 and March 2019, and they were enrolled in the study (Figure 1).

Of these patients, 167 were lost to follow-up and therefore excluded from analysis, leaving 334 patients with either a minimum of 6 months follow-up or with a diagnosis of GA within 6 months of injury. Two of the patients who returned at 6 months or later for clinical review completed questionnaires but did not have radiographs available and were therefore excluded from our analysis. Of the 332 patients with adequate follow-up (mean 16.1 months, standard deviation [SD] 10.0 months), 100 (30.1%) had evidence of GA on follow-up radiographs. Demographics of each of these three groups are summarised in Table 1. Across all 332 patients included in the analysis, mean age at time of injury was 10.4 years (SD 3.7 years), 204 (61.4%) were male, and 159 (47.9%) injuries were on the right limb. The SH classification is recorded in Table 2. The majority of fractures were SH type 2 with no SH type 5 fractures identified throughout the duration of the study.

Rates of agreement between the two orthopaedic surgeons (NH and LJ) were 85.8% with a Cohen’s Kappa statistic of 0.63 (95% Confidence Interval [CI] 0.53, 0.72).

Patients who developed GA were more likely to be closer to skeletal maturity (12.8 years vs. 9.4 years) and injured on the right side (53.0% vs. 45.7%). These patients were also more likely to require operative fixation (20.0% versus 8.6%). Those in the GA group had higher rates of initial displacement requiring reduction (62.0% versus 45.7%). There were higher rates of angulation in the GA group (47.0% vs. 38.8%), but the amount of angulation was similar (27.0° vs. 27.0°). These results are summarised in Table 1. More patients who went on to develop GA had a high energy injury at 24.0% compared with 8.2% in the group without GA. High-energy injuries were defined as a fall greater than three metres, motor vehicle accidents, contact sports or a similar mechanism. Of those who developed GA, 75% had an SH type 2 fracture.

When investigating risk factors for growth arrest, we found that age, requiring reduction and operative fixation all significantly increased the odds of developing growth arrest (Table 3).

Fracture location has been summarised in Table 4. The most commonly affected bone was the radius, accounting for 141 (42%) fractures, 118 of which were distal radial fractures, 32 (27%) of which went on to develop GA. Rates of GA were highest in distal femoral fractures (86%), proximal tibia (50%), distal tibia (45%) and proximal humerus (43%). Patients with a fracture in the distal femur had the greatest odds of developing GA (OR 16.13, 95% CI 2.62, 310.87).

During the course of the study, 12 patients required further intervention for GA. Six patients underwent an epiphysiodesis of either the contralateral limb to equalise leg lengths or the distal ulna or fibula to prevent further deformity due to disparate growth following GA. Of the remaining six, two patients had physeal bar resections, two underwent guided growth, one had an ulnar shortening osteotomy, and one underwent serial splinting to improve the range of motion.

## 4. Discussion

This study of paediatric physeal fractures is the largest prospective cohort to our knowledge and has shown a higher than previously reported rate of GA at 30.1%. This increased rate could be due to differences in the threshold criteria for diagnosing a GA. The rates of GA were higher in lower extremity injuries at 43% (52 patients). This is consistent with previous studies, which have also reported higher rates of GA in lower extremities [2,15,16].

The distal femur is particularly vulnerable, with GA occurring in 86% of our study population; however, distal femoral fractures were uncommon, accounting for only 8 out of the 332 fractures (2.4%). This finding is consistent with the existing literature; a 2009 meta-analysis of 564 fractures found GA accorded in 36–64% of distal femoral fractures, depending on the SH type [17]. A retrospective study by Arkader et al. focusing on distal femoral fractures also reported a high rate of GA at 27% (20 patients); however, it was only clinically significant in 11 of these patients, 9 of whom had significant displacement on presentation. They found that the degree of initial displacement and SH classification strongly correlated with outcomes, particularly that of GA [18].

Tibial injuries also seem to be prone to GA with 45% of distal and 50% of proximal tibial fractures resulting in GA in the study cohort. This is a substantively higher rate than the 12.8% seen in a retrospective series of 78 distal tibial fractures [19]. The study found initial displacement to be the only significant risk factor in the development of GA. More in line with our findings, a retrospective review of distal tibial physeal injuries by Barmada et al. found a rate of premature physeal closure of 33% with over half (64%) of patients requiring further surgery to accommodate GA [20]. They also saw a high incidence of GA in SH type 1 and 2 fractures at 36% with a 3.5-fold increased risk of GA when a physeal gap was present on post-reduction films in SH type 1 and 2 fractures. Barmada et al. advocate for the use of open reduction of these fractures to decrease the risk of GA.

This is in contrast to other studies demonstrating higher rates of GA with SH type 4 and 5 fractures [9]. In a rat model, Wattenbarger et al. found physeal bars to be associated with the fracture line extending through the whole physis. They also showed that the plane of the fracture plays a significant role, with an extension into the physeal–epiphyseal border resulting in higher rates of cellular disorganisation and subsequent bar formation which may explain higher rates of physeal arrest in SH type 3 and 4 fractures reported previously [21]. Our study does not appear to support this hypothesis, with the highest rate of GA in the SH type 2 group at 32.9% (75 fractures). In comparison, GA was seen in 29.4% of SH type 1 fractures, 25.9% of SH type 3 fractures, and 21.7% of SH type 4 fractures.

SH type 2 fractures are the most commonly seen physeal injuries accounting for 228 (68.7%) of physeal fractures in our study [5]. The higher incidence of GA in SH type 2 fractures may have been influenced by the way these fractures were classified; in particular, lateral condyle fractures were classified as SH type 4 as per Salter and Harris’ original paper [9]. The classification of these fractures has been shown to have poor correlation between pre-operative and intra-operative findings, and it may be that a proportion of these were actually SH type 2 fractures [22]. Lateral condyle fractures result in a low rate of GA and reclassifying them as SH type 2 fractures may alter the rate of GA observed per SH fracture type. Similarly, distal tibial fractures may also require further advanced imaging to accurately determine the SH classification; this is particularly significant given the high rate of physeal arrest (45%) demonstrated in distal tibial fractures in our study. A similar finding was noted by Mann et al., who recommended advanced imaging in these fractures in order to accurately define and classify the fracture pattern [4].

Age at time of injury appears to be strongly associated with rates of GA, with an average age of 12.8 years in the GA group compared to 9.4 years in the group without GA. This may be due to inherently higher energy injuries in older children due to larger body mass and participation in high energy contact sports, resulting in more force going through the physis. High-energy injuries were more common in the GA group at 24.0% compared to 8.2% in the group without GA. As adolescents approach skeletal maturity, there may also be changes in the physis that influence its susceptibility to injury. Our review of the current literature suggests that there are no published studies observing this phenomenon. As children approach skeletal maturity the impact and clinical relevance of GA may be diminished. This is reflected in the low incidence of operative intervention within our GA group.

This study has demonstrated a trend towards increased rates of GA or bar formation with residual displacement and further reduction attempts. Of the patients who developed GA, 62.0% required reduction compared to 45.7% of those who did not develop GA. In a recent study, the risk of GA was 11% after one reduction attempt, with observed increases to 24% after a second reduction attempt and even further up to 50% after a third reduction attempt [23]. The rates of angulation in the GA group were higher, but the actual amount of angulation was similar (27.0° versus 28.4°), suggesting displacement is more important than angulation in predicting GA.

Limitations of this study include the use of plain films to determine GA. MRI and CT were utilised at the treating surgeons’ discretion but were not ordered routinely for follow-up due to concerns regarding unnecessary cost and radiation exposure, respectively. A study looking at MR imaging of GA showed that radiographs were adequate to assess GA in the majority (72/111) of patients, with the earliest bone bridge present at two months post-injury [16]. Our use of plain films may have resulted in missed physeal bars that could have been apparent on MRI, which would result in an even higher rate of GA. Small bars not evident on plain film are, however, unlikely to be clinically significant, as any resultant angular deformity would have been recognised on plain film analysis. However, it is also worth noting that the standard of care in clinical practice to assess healing after fractures, and to look for evidence of GA, is the use of plain radiographs. While MRIs may provide more sensitive and detailed information, they are not commonly used in clinical practice and come with additional costs that would limit their use. MRIs or more advanced 3D MRIs may instead have more utility in guiding pre-operative planning for confirmed GA [24,25].

Another limitation of this study was a large rate of loss to follow-up, despite the prospective nature of the study. Loss to follow-up is a recognised but under-reported problem in orthopaedic trauma [26], particularly in paediatric populations. Previous studies in adult trauma populations have cited socioeconomic factors, demographic factors such as gender and health behaviours and insurance status as factors in follow-up. A Canadian study found patients lost to follow-up tended to be from marginalised groups and/or had accessibility barriers to attending clinics [27]. Given the potential role of social factors influencing follow-up, it is possible that our loss to follow-up group is biased toward lower socioeconomic status or minority ethnic groups who may have increased barriers to accessing care. However, we did not collect data on these factors, and this would be an interesting future avenue to explore. Patients in the loss to follow-up group were, on average, slightly older (10.5 years vs. 9.4 years) than those who completed follow-up but did not develop GA but still younger than those who went on to develop GA (12.8 years). Reduction was required less frequently in this group (40.5%) than those who completed follow-up and did or did not develop GA (62.0% and 45.7%, respectively). None of the patients in the loss to follow-up group had fixation across the physis compared to 70.0% in the GA group, and those lost to follow-up had fewer high-energy injuries (10.8% vs. 24.0%), suggesting that the risk of GA in the loss to follow-up group was low. Consequently, parents may have been less likely to feel the need to attend follow-ups if their child was doing well. This study was performed at the regional tertiary referral centre so it is likely anyone who developed GA and presented elsewhere would be referred for ongoing management. However, we cannot exclude the possibility that some patients developed GA and were managed at an outside institution. There was a reasonably high rate of early loss to follow-up, with only 78.6% of those lost attending their 6–8 week follow up, and even less (37.5%) attending follow-up at 4–8 months. A 2020 study examining “no-shows” in a paediatric orthopaedics clinic found that a longer length of time between scheduling of the appointment and the appointment itself was a predictive factor [28]. This could have been a contributor to the loss to follow-up we observed at 4–8 months and later time points.

Cessation of growth may not occur immediately after a physeal injury; there can be a delay of up to 6 months, and GA may be preceded by a period of slowing growth [9]. Of those who developed GA, 78/100 had data showing the point at which the possibility of GA was initially raised, 75.6% (59/78) of which were before eight months of follow-up. This suggests follow-up to eight months is advisable but ongoing follow-up beyond that point may be on an as-needed basis, or for those with a high index of suspicion or risk factors for GA [29].

## 5. Conclusions

In conclusion, this study has demonstrated higher than expected rates of GA at 30.1%. GA was found to be more common in older children and lower limb fractures, with the majority of GA noted to be asymptomatic requiring no further intervention. As some fracture patterns, such as distal femoral fractures, are relatively rare, a larger, multi-centre study is required in order to accurately create and validate a clinical prediction model that takes into account fracture type and key patient demographics. Such a validated prediction model would further guide patient management in both the acute and long-term follow-up settings. Additionally, advanced imaging studies on X-ray-identified GA could help further define features of GA that could predict future prognosis. Finally, long-term follow-up will be required to understand the clinical significance and implications of a GA at 6 months post-injury in different age groups of the paediatric population.

## Figures and Tables

**Table 1:** Baseline demographics.

Variable	No Growth Arrest (N = 232)	Growth Arrest Present (N = 100)
**Age in years at baseline, mean (SD)**	9.4 (3.7)	12.8 (2.6)
**Male sex, N (%)**	142 (61.2)	62 (62.0)
**Right side, N (%)**	106 (45.7)	53 (53.0)
**Fracture angulated, N (%)**	90 (38.8)	47 (47.0)
**Degrees of angulation, mean (SD)**	28.4 (21.9)	27.0 (21.8)
**Fracture displaced, N (%)**	111 (47.8)	59 (59.0)
**Millimetres displaced, mean (SD)**	6.2 (4.7)	7.5 (5.7)
**Required reduction, N (%)**	106 (45.7)	62 (62.0)
**Operative fixation, N (%)**	20 (8.6)	20 (20.0)
**Fixation across physis, N (%)**	12 (60.0)	14 (70.0)
**Energy of Injury, N (%)**		
**High**	19 (8.2%)	24 (24.0%)
**Moderate**	170 (73.2%)	58 (58.0%)
**Low**	40 (17.2%)	16 (16.0%)

**Table 2:** Salter–Harris (SH) classification by growth arrest.

SH Fracture Type	Total (N = 332)	No Growth Arrest (N = 232)	Growth Arrest Present (N = 100)	Odds Ratio (95%CI); *p*-Value
**SH Type 1**	17 (5.1%)	12 (70.6%)	5 (29.4%)	Reference
**SH Type 2**	228 (68.7%)	153 (67.1%)	75 (32.9%)	1.18 (0.42, 3.83); 0.77
**SH Type 3**	27 (8.1%)	20 (74.1%)	7 (25.9%)	0.84 (0.22, 3.40); 0.80
**SH Type 4**	60 (18.1%)	47 (78.3%)	13 (21.7%)	0.66 (0.20, 2.39); 0.51

Adjusted for age, sex, fracture location, reduction required, and fixation required.

**Table 3:** Possible risk factors for growth arrest.

Variable	Crude Odds Ratio (95%CI); *p*-Value	Adjusted * Odds Ratio (95%CI); *p*-Value
**Age in years at baseline**	1.41 (1.28, 1.56); <0.001	1.62 (1.42, 1.88); <0.001
**Required reduction**	1.89 (1.18, 3.08); 0.009	1.46 (0.74, 2.93); 0.27
**Operative fixation**	2.65 (1.35, 5.20); 0.005	3.93 (1.49, 10.90); 0.007

* Adjusted for sex, SH classification and fracture location.

**Table 4:** Fracture location.

Fracture Location	Developed Growth Arrest	Crude Odds Ratio * (95%CI); *p*-Value
**Femur (n = 8)**	6 (75%)	
**Proximal (n = 1)**	0	0 (NA, 0); 0.99
**Distal (n = 7)**	6 (86%)	16.13 (2.62, 310.87); 0.011
**Fibula (n = 23)**	5 (22%)	
**Proximal (n = 0)**	0	NA
**Distal (n = 23)**	5 (22%)	0.75 (0.23, 2.05); 0.59
**Humerus (n = 62)**	10 (16%)	
**Proximal (n = 7)**	3 (43%)	2.02 (0.38, 9.63); 0.38
**Distal (n = 55)**	7 (13%)	0.39 (0.15, 0.91); 0.039
**Radius (n = 141)**	37 (26%)	
**Proximal (n = 23)**	5 (22%)	0.75 (0.23, 2.05); 0.59
**Distal (n = 118)**	32 (27%)	Reference
**Tibia (n= 90)**	41 (46%)	
**Proximal (n = 12)**	6 (50%)	2.69 (0.79, 9.19); 0.11
**Distal (n = 78)**	35 (45%)	2.19 (1.20, 4.02); 0.011
**Ulna (n = 8)**	1 (13%)	
**Proximal (n = 1)**	0 (0%)	0 (NA, 0); 0.99
**Distal (n = 7)**	1 (14%)	0.45 (0.02, 2.76); 0.47

* Reference group is distal radius.

## Data Availability

Aggregate data presented in this study could be made available upon reasonable request due to privacy and ethical reasons.
